# Gait Speed Predicts Response to Physical Activity Promotion in Older Adults with COPD

**DOI:** 10.1093/geroni/igaf122.2740

**Published:** 2025-12-31

**Authors:** Patricia Bamonti, Stephanie Robinson, Marilyn Moy

**Affiliations:** VA Boston Health Care System, Boston, Massachusetts, United States; VA Bedford Health Care System, Bedford, Massachusetts, United States; VA Boston Health Care System, Boston, Massachusetts, United States

## Abstract

Physical activity (PA) interventions are efficacious in individuals with chronic obstructive pulmonary disease (COPD), but not all participants improve to the same degree. Identifying characteristics that predict response to PA interventions can inform future intervention development. Gait speed is a useful indicator of physical performance, but it is unknown whether it predicts response to PA intervention. We examined whether baseline gait speed predicted response to a PA intervention. Secondary analyses included two cohorts of U.S. Veterans with COPD (N = 163 with complete data). Data from a web-based, pedometer-mediated PA intervention group (n = 89) and a control group (PA and disease self-management with or without a pedometer; n = 74) were combined. Participants completed a 243-meter walking course at their usual gait speed. Those who achieved a minimally clinically important difference (MCID) of 1000 average daily steps/day improvement from baseline to 3-month follow-up were categorized as “responders.” A logistic regression model tested the association of gait speed and odds of being a responder, after controlling for age, FEV1 % predicted, baseline average daily steps (over 7-10 days), season, group assignment, and study. Participants (98% male; mean age 69±8 years) had baseline mean gait speed of 1.09±.20 m/s, and 28.8% were responders. For every 0.10 m/s increase in gait speed, equivalent to the MCID for gait speed in COPD, the odds of achieving the MCID was 34% greater (adjusted odds ratio=1.34, 95% CI [1.07, 1.70]). Baseline gait speed can inform targeted selection of individuals most likely to benefit from PA interventions.

